# Meteorological Report for June

**Published:** 1880-07

**Authors:** 


### METEOROLOGICAL REPORT FOR JUNE, 1880.

| RAIN. DATE | IN. | Mean daily Barometer. | Mean daily Thermometer | Mean daily Humidity. | Maximum Thermometer | Minimum Thermometer | Prevailing direction of Wind. | Maximum hourly velocity of Wind, (Daily.) |
|---|---|---|---|---|---|---|---|---|
| 1 | 25 | 29.972 | 77.2 | 78.7 | 86 | 73 | W. | ......... |
| 2 | 0 | 29.777 | 72.7 | 75.7 | 82 | 69 | N. W. | ......... |
| 3 | 0 | 30.118 | 69.2 | 61.3 | 82 | 63 | E. | ......... |
| 4 | 0 | 30.138 | 68.7 | 52.0 | 76 | 60 | E. | ......... |
| 5 | 0 | 30.082 | 71.2 | 66.0 | 79 | 61 | E. | ......... |
| 6 | 0 | 29.944 | 75.5 | 69.7 | 82 | 65 | S. | ......... |
| 7 | 1.16 | 29.935 | 73.7 | 79.7 | 84 | 69 | S. W | ......... |
| 8 | 01 | 29.951 | 77.0 | 64.0 | 84 | 67 | N. W. | ......... |
| 9 | 0 | 29.991 | 81.5 | 60.7 | 88 | 67 | N. E. | ......... |
| 10 | 12 | 30 047 | 84.2 | 58.3 | 89 | 74 | S. | ......... |
| 11 | 85 | 30.086 | 80.2 | 65.7 | 90 | 72 | N. W. | ......... |
| 12 | 0 | 30.013 | 84.5 | 43.7 | 91 | 72 | N. W. | ......... |
| 13 | 10 | 29.940 | 84.7 | 59.0 | 92 | 78 | W. | ......... |
| 14 | 0 | 29.850 | 85.0 | 58.3 | 92 | 78 | W. | ......... |
| 15 | — | 29.753 | 80.7 | 65.7 | 90 | 75 | W. | ......... |
| 16 | 0 | 29.890 | 69.7 | 60.7 | 80 | 66 | N. W. | ......... |
| 17 | 0 | 30.059 | 70.0 | 42.0 | 78 | 60 | E. | ......... |
| 18 | 0 | 30 169 | 71.0 | 39.0 | 78 | 58 | N. E. | ......... |
| 19 | 0 | 30.172 | 71.7 | 45.3 | 79 | 62 | N. E. | ......... |
| 20 | 0 | 30 091 | 74 0 | 43.3 | 81 | 62 | E. | ......... |
| 21 | 0 | 30.007 | 75.5 | 43.0 | 81 | 64 | E. | ......... |
| 22 | 0 | 29.961 | 78.0 | 49.0 | 85 | 67 | E. | ......... |
| 23 | — | 29.965 | 77.0 | 64.7 | 87 | 68 | S. E. | ......... |
| 24 | 02 | 29.979 | 76.7 | 71.0 | 84 | 69 | S E. | ......... |
| 25 | 97 | 30.041 | 73.2 | 86.7 | 80 | 71 | S. | ......... |
| 26 | 07 | 30.105 | 76.7 | 74.7 | 86 | 70 | S. | ......... |
| 27 | 0 | 30 085 | 82.2 | 61.3 | 89 | 71 | S. W. | ......... |
| 28 | 0 | 30.021 | 83.0 | 56.3 | 89 | 72 | S. W. | ......... |
| 29 | 0 | 29.972 | 81.2 | 59.3 | 87 | 74 | S. W. | ......... |
| 30 | 02 | 30.058 | 80.7 | 64 0 | 88 | 71 | W. | ......... |
| ...... | ...... | .......... .. | ............ | ............ | ......... | ......... | ......... | ......... |
| Sums | 4.52 | 30.012 | 76 9 | 60.6 | 84.6 | 68.3 | ......... | ......... |

Mean monthly barometer, 30 012.
Highest Barometer, 30.245, on the 19th.
Lowest Barometer, 29.710, on the 15th.
Monthly range of Barometer, 0.535.
Highest Temp. 92°, on 13th and 14th. Lowest Temp. 58°, on 18th.
Monthly range of Temperature, 34°.
Greatest daily range of Temperature, 21°, on the 9th.
Least daily range of Temperature, 9°, on the 25th.
Mean of Maximum Temperatures, 84°.6
Mean of Minimum Temperatures, 68°.3
Mean daily range of Temperature, 15°7. Total rainfall, 3.57 inches.
Prevailing wind, E. Total movement of wind, 16.69 miles.
Maximum velocity of wind and direction, 28 miles per hour, on the 10th.
Number of cloudy days on which rain fell, 5.
Number of cloudy days on which no rain fell, 3.
Total number of days on which rain fell, 11.

H. HALL, Corp'l. Signal Service U. S. A.

Atlanta, Ga., June, 1880.

